# Morphological diversity of dental and mandibular deformities in *Microtus hartingi* (Rodentia, Arvicolinae)

**DOI:** 10.1093/cz/zoaf052

**Published:** 2025-08-13

**Authors:** Tanya Zorenko, Anton Basov, Ugis Kagainis, Fedor Golenishchev

**Affiliations:** Faculty of Medicine and Life Sciences, University of Latvia, 1 Jelgava Street, Riga LV-1004, Latvia; Faculty of Medicine and Life Sciences, University of Latvia, 1 Jelgava Street, Riga LV-1004, Latvia; Institute of Biology, University of Latvia, 4 O. Vaciesa Street, Riga LV-1004, Latvia; Russian Academy of Sciences, Zoological Institute, Universitetskaya Emb. 1, Saint Petersburg 199034, Russia

**Keywords:** compensatory mechanism, *Microtus hartingi*, geometric morphometrics, mandible, incisor overgrowth, vole

## Abstract

We analyzed 104 labial and lingual projections of Harting's vole (*Microtus hartingi*) mandibles using linear and geometric morphometrics. Incisor and molar overgrowth and mandibular deformations were described in 2 Harting's vole populations. Over a 6-year research period, excessive incisor growth was observed in 15.4% of the Rhodopean *M. hartingi* population and in 10.3% of the Central Anatolian form *M. h. ankaraensis*. Maxillary incisors lengthening up to 2.3× the norm was noted, often accompanied by mandibular incisor shortening. Nevertheless, voles with overgrown incisors were able to feed and breed until the late stages of the pathology, where overgrown incisors made feeding impossible. The mandibular deformations were always accompanied by incisor overgrowth and never manifested on their own. Geometric morphometrics analysis revealed similar patterns of mandibular deformation and incisor overgrowth in both vole populations: extension of the coronoid process and the body of the mandible, elevated molar alveolus, deformation of the incisor alveolus, shortening of the diastema and narrowing of the spaces between the mandibular processes. Linear morphometry did not reveal any significant changes in the mandibular morphoecological indices; however, the distances between landmarks changed significantly. Due to the mandibular shape change, the methiorphoecological index values of the deformed mandibles remained the same as those of the normal mandibles. The excessive tooth growth and deformed mandible morphotype in Harting's vole may be the result of genetic and epigenetic factors induced by environmental stress which lead to developmental anomalies.

## Graphical Abstract

**Graphical Abstract:**
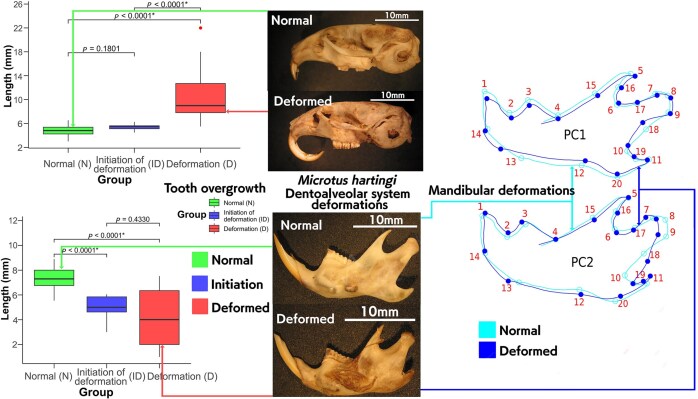

The rodent dentoalveolar system responds subtly to environmental factors throughout evolution (Renvoisé et al. 2009; Jheon et al. 2015; Kovaleva et al. 2021) and in real time within a population. Variability in the shape parameters of the cranium, mandible and teeth provides information about the state of the population, reflecting the response of animals to constantly changing abiotic (Zakharov et al. 2000; Söderman et al. 2007) and biotic factors (e.g., fragmented habitat, social stress, inbreeding) (Zorenko 2022; Zorenko and Kaija 2024). These factors, being stressors, inevitably disrupt developmental homeostasis, primarily affecting morphological structures, which leads to phenotypic diversity (Zakharov and Yablokov 1997). Ecological stress effects may be enhanced in the presence of genetic stress (e.g., inbreeding, mutations, genomic coadaptation disruption) (Parsons 1992).

In voles, changes in the structure and shape of teeth and mandibles are observed both in nature (Jentzsch et al. 2020) and in laboratory colonies (Imai et al. 2018; Zorenko et al. 2025). The most common condition is mandibular and maxillary molar elongation. In addition, protrusion of the mandibular molar alveolar bone and the penetration of maxillary molar apices into the cranial cavity have been noted in many vole species (Sugita et al. 1995; Imbschweiler et al. 2011; Jheon et al. 2015; Imai et al. 2018; Golenishchev et al. 2022). In the pine vole (*Mynomes pinetorum*), unlike other vole species, coronal molar elongation, rather than apical elongation, has been observed (Harvey et al. 2009). Maxillary incisor elongation in voles is less common (Harvey et al. 2009; Imbschweiler et al. 2011; Jheon et al. 2015; Jentzsch et al. 2020), and ranges from slight (Gill and Bolles 1982 ; Imai et al. 2018) to significant incisor curvature (Zorenko 2021).

Few studies have focused on mandibular deformations; however, such changes have been noted in several works: bone proliferation, deformation, and bone mass loss were described in the common vole (*Microtus arvalis*) and in the short-tailed field vole (*Euarvicolla agrestis*) (Jentzsch et al. 2020). Severe mandibular alveolar bone deformation due to protrusion of the molar apices was observed in several other vole species (Gill and Bolles 1982; Jheon et al. 2015; Imai et al. 2018). Congenital malocclusion has been observed in several rodent species: the steppe lemming (*Lagurus lagurus*) (Imbschweiler et al. 2011), the long-tailed vole *(My. longicaudus*) (Maser and Hooven 1970), the pine vole (Harvey et al. 2009), and the golden hamster (*Mesocricetus auratus*) (Mancinelli and Capello 2016). Various hypotheses attempt to explain the occurrence of bone deformation in laboratory rodent colonies. These include a soft diet (lack of solid materials suitable for tooth abrasion), osteomyelitis, development of odontogenic tumors and tumor-like lesions (odontomas), genetic factors, or trauma caused, for example, by chewing on the iron cage bars (Mancinelli and Capello 2016).

In healthy animals, the shape and biomechanical parameters of the mandible are primarily determined by the diet type (Anderson et al. 2014). Experimental studies on several rodent species have shown that animals fed a soft diet had shorter mandibles, smaller jaw adductor muscles, and smaller muscle attachment areas (Engström et al. 1986; Yamada and Kimmel 1991). However, the nutrition may also affect the mandible size, whereas shape is determined solely by mechanical strain. Mandibles of rats fed a low-calcium diet were smaller in all dimensions, whereas rats fed a soft diet regardless of calcium content had a reduced mandibular vertical height and condyle size (Lieberman et al. 2004). In addition, a soft diet weakens the masticatory muscles resulting in a bone mineral density decline (Mavropoulos et al. 2005).

Many authors suggest that dentoalveolar system deformations have a hereditary basis (Gill and Bolles 1982; Ida-Yonemochi et al. 2002; Imbschweiler et al. 2011; Jheon et al. 2015; Imai et al. 2018). Various genes affect the development of both teeth and mandibles simultaneously. For example, RUNX2 gene-deficient mice lacked mandibular bone and exhibited tooth eruption arrest (James et al. 2006; Ma et al. 2018). Gene mutations responsible for malocclusion formation and shortening of molars and incisors were observed in the inbred mice strains (Grϋneberg 1963; Jheon et al. 2015). A non-Mendelian inheritance pattern and synergistic interaction between genetic and environmental factors were demonstrated (Gill and Bolles 1982). It has been assumed that apical molar elongation in the prairie vole *(My. ochrogaster*) could be induced by a spontaneous mutation, resulting in the dental stem cell niche misregulation (Jheon et al. 2015).

The mandible is the only movable bone of the rodent's skull. It acts as a lever, articulating with the temporal bone of the skull by the condylar process. The structure of the mandible ensures strong compression of the incisors during gnawing while also allowing for grinding movements for chewing. Voles, predominantly herbivorous rodents, use a special chewing method—longitudinal grinding, based on longitudinal rather than transverse displacements of the mandible, unlike rabbits and ruminants. This method is characteristic of hypselodont molars, which have a flat crown and cutting elements in the form of enamel ridges. Cutting occurs within a very narrow contact zone between the enamel ridges of the mandibular and maxillary molars (Potapova 2020).

A vole's mandible is short and relatively tall, with a long diastema for incisor accommodation, a high articular head and a short coronoid process. The articular head is usually elongated longitudinally, and the articular fossa has a trough-shaped form (Potapova 2020). Weijs (1975) in a study of albino rats (Wistar strain) showed that these species have only the bilateral symmetric chewing mechanism; however, lateral asymmetry (lateralization) of the mandible is the most important feature of mammalian dentoalveolar system (Nikol'sky 1997; Zamanlu et al. 2012; Parés-Casanova and Bravi 2014; Ginot et al. 2018). In voles, the mandible consists of 2 parts connected by a flexible fibrocartilaginous symphysis, allowing the 2 hemimandibles to move more independently from each other. The flexibility of the hemimandibles in the symphyseal region not only in the anteroposterior direction but also laterally suggests the possibility of independent chewing movements in voles, albeit with the low amplitude (Gromov and Polyakov 1977). Gromov proposes that in voles the food grinding might be possible not only with the whole jaw, but also alternately on different sides of the jaw.

Simultaneous bilateral occlusion is impossible as the food processing occurs on the working side of the mandible, whereas the other side serves as a balance. The chewing process is coordinated by the 2 adductor muscles (m.masseter and m.temporalis), which determine the direction of the mandibular movements (Nikol'sky 1997). This allows the animal to chew by using only 1 hemimandible, leaving the other hemimandible less loaded. Although the voles' mandible is an integrated structure, hemimandible flexibility in the area of the symphysis allows for separate chewing movements, albeit with a limited amplitude. This is indirectly evidenced by the unequal wear of the mandibular molar surfaces on each hemimandible. However, this is not experimentally proved (Gromov and Polyakov 1977).

Mandibular ontogenesis is a complex and lengthy process. It begins in the embryonic period and continues after birth, lasting until the onset of puberty. All mandibular components (the ramus, molar and incisor alveoli, the coronoid, condylar, and angular processes) are present from embryonal day 17 in mice and voles (Štebra 1976; Martinez-Maza et al. 2012). During this developmental period, the impact of stress can be significant (Kaija et al. 2024).

Voles have 1 pair of hypselodont incisors and 3 pairs of hypselodont molars. They have to constantly chew and gnaw to maintain the proper morphology of their ever-growing teeth (Müller et al. 2014a, 2014b). The non-involvement of molars in the gnawing process results in uncontrolled growth and changes in the morphology (Gill and Bolles 1982; Imai et al. 2018). Voles' ever-growing incisors and molars house stellate reticulum cells located between the inner and outer enamel epithelium. The stellate reticulum and outer enamel epithelium of the molar region and the labial cervical loop of the incisor contain stem cells that promote continuous tooth growth (Goedendorp et al. 2013; Jheon et al. 2015). These stem cells are involved in both the regulation of tooth eruption and bone formation. Bone morphogenetic proteins can induce the tooth's follicle cells proliferation into osteogenic cells (Zhou et al. 2019; He et al. 2021). Mesenchymal stem cells form the mandibular components. These cells also can differentiate into odontoblasts, which produce dentin, forming the dentin layer of the tooth. Formation processes are similar in both hypselodont incisors and hypselodont molars (Ohshima et al. 2005; Liu et al. 2010; Kaku and Yamauchi 2014; He et al. 2021), although some differences may exist (Jheon et al. 2015).

In Harting's vole (*M. hartingi*) from the Eastern Rhodopes (Mandrica, Bulgaria), incisor overgrowth was observed in some animals soon after they were captured in the wild, and later in the laboratory in the F1–F6 generations. Mandibular deformations were discovered later and were always accompanied by dental abnormalities, but never appeared on their own (our data). In the Anatolian (Kırşehir province, Central Anatolia, Turkey) population (*M. h. ankaraensis*), heterochrony and mandible deformation began to appear only after the exposure to experimental social stress caused by the formation of communal groups, as well as physical stress to which pregnant and lactating females were exposed (Zorenko 2022). The Anatolia vole *M. h. ankaraensis* has been assigned subspecies status (Yiğit et al. 2017), whereas the taxonomic status of the Rhodopean *M. hartingi* population remains unclear (Golenishchev et al. 2022).

The aim of this research was to study abnormalities in the structure of the mandible and teeth of 2 Harting's vole populations from the Eastern Rhodopes and from Central Anatolia. Our objectives were: 1) to assess morphological changes in the incisors and molars, 2) to assess the deformation patterns of the mandible using linear (LM) and geometric morphometrics (GM), and 3) to determine whether mandibular deformations serve as a compensatory mechanism that allows Harting's voles with dental growth anomalies to feed and reproduce.

## Materials and methods

### Morphometry

#### Model object

*M. hartingi* laboratory population founders were sampled in 2 locations: Rhodopean population *M. hartingi* (“*HarR*”: Eastern Rhodopes, Mandrica, Bulgaria: 41°41′N, 26°12°′E) and Central Anatolian population *M. h. ankaraensis* (“*Ank*”: Kırşehir province, Central Anatolia, Turkey: 39°9′N 34°6′E). For the GM and linear morphometry analysis we examined the mandibles of 56 *HarR* (25 females and 31 males), of which 9 specimens were collected from the wild and the rest from DEZ laboratory colony (collection of the department of Zoology, University of Latvia), and 50 mandibles of *Ank* (24 females and 26 males), of which 19 specimens photos were obtained from ZIN (Zoological Institute, Russian Academy of Sciences) collection and the rest from DEZ laboratory colony.

The mandibles of *HarR* were separated into normal (N) and deformed (D) groups. The mandibles of *Ank* from DEZ were decided as initiation of deformation (ID) and deformed (D). Photos from ZIN were marked as normal (N). The normal group had no visible mandibular deformations and maxillary incisors were shorter than mandibular incisors. The initiation of deformation was considered for animals, in which maxillary and mandibular incisors were becoming 1 length and the mandible is slightly deformed or not deformed. Deformed group had noticeably deformed mandibles and overgrown maxillary incisors. The average age of *HarR* individuals was 6.8 months (2–9) for N and 5.4 months (2–9) for D, and of *Ank*—5.3 (2–7) for ID and 4.2 (2–7) months for D, the N age data were not available.

#### Microscopy

Nineteen left side mandibles of *Ank* from ZIN collection were photographed in lateral view using stereomicroscope equipped with Canon EOS 60D camera and further processed in Helicon Focus v. 6.8.0 and Helicon Remote v. 3.9.2W. Canon Photo Shot S70 camera was used to obtain pictures of 88 mandible specimen sets from DEZ (Table 1). In each set, 4 images were obtained: labial and lingual views for both left and right hemimandibles. During the photography, all specimens were fixed separately on a movable piece of plasticine (height: 10 mm), so the incisor plane was parallel to the plane of the table. A millimeter scale was placed near each specimen during the photography for further scale calibrations. Appropriate magnification and focal length (4 cm) were chosen for visibility of an entire specimen in a single field of view. The population, deformation degree, sex, view position (labial or lingual), and 3-digit code of an individual ID were coded in the name for each digital photograph (digital resolution: 4272 · 2848 pixels for ZIN and 3072 · 2304 pixels for digital images of DEZ specimens).

**Table 1:** Numbers of specimens among 4 sets used in statistical analysis of different vole subspecies.

		N	ID	D	N	ID	D
Population	Dataset	Females			Males		
*HarR*	Labial (left)	15	NA	9	11(3)	NA	17(1)
	Labial (right)	14(1)	NA	9	14	NA	18
	Lingual (left)	15	NA	9	14	NA	17(1)
	Lingual (right)	15	NA	8(1)	14	NA	17(1)
*Ank*	Labial (left)	10	2	10	9	14	5(1)
	Labial (right)	NA	2	7(3)	NA	14	5(1)
	Lingual (left)	NA	1(1)	10	NA	13(1)	5(1)
	Lingual (right)	NA	2	9(1)	NA	14	5(1)

*HarR*, *Microtus hartingi*; *Ank*, *Microtus h. ankaraensis*; N, normal; ID, initiation of deformation; D, deformation; NA, no specimens in the referred group; numbers of excluded specimens during statistical analysis of GM are indicated in parentheses.

#### Tooth length analysis

Tooth length was manually measured using calipers (Topex). The measurement was made from the edge of the incisor alveolus to the tip of the incisor. Additional measurements of incisor length obtained over the 6 years of colony existence were included in the analysis. To characterize mandibular shape changes, parameters such as the length of the upper diastema and lengths of the maxillary and mandibular molars (M1–M3, and m1–m3, in mm) were obtained.

### GM and shape analyses

**Figure 1:**
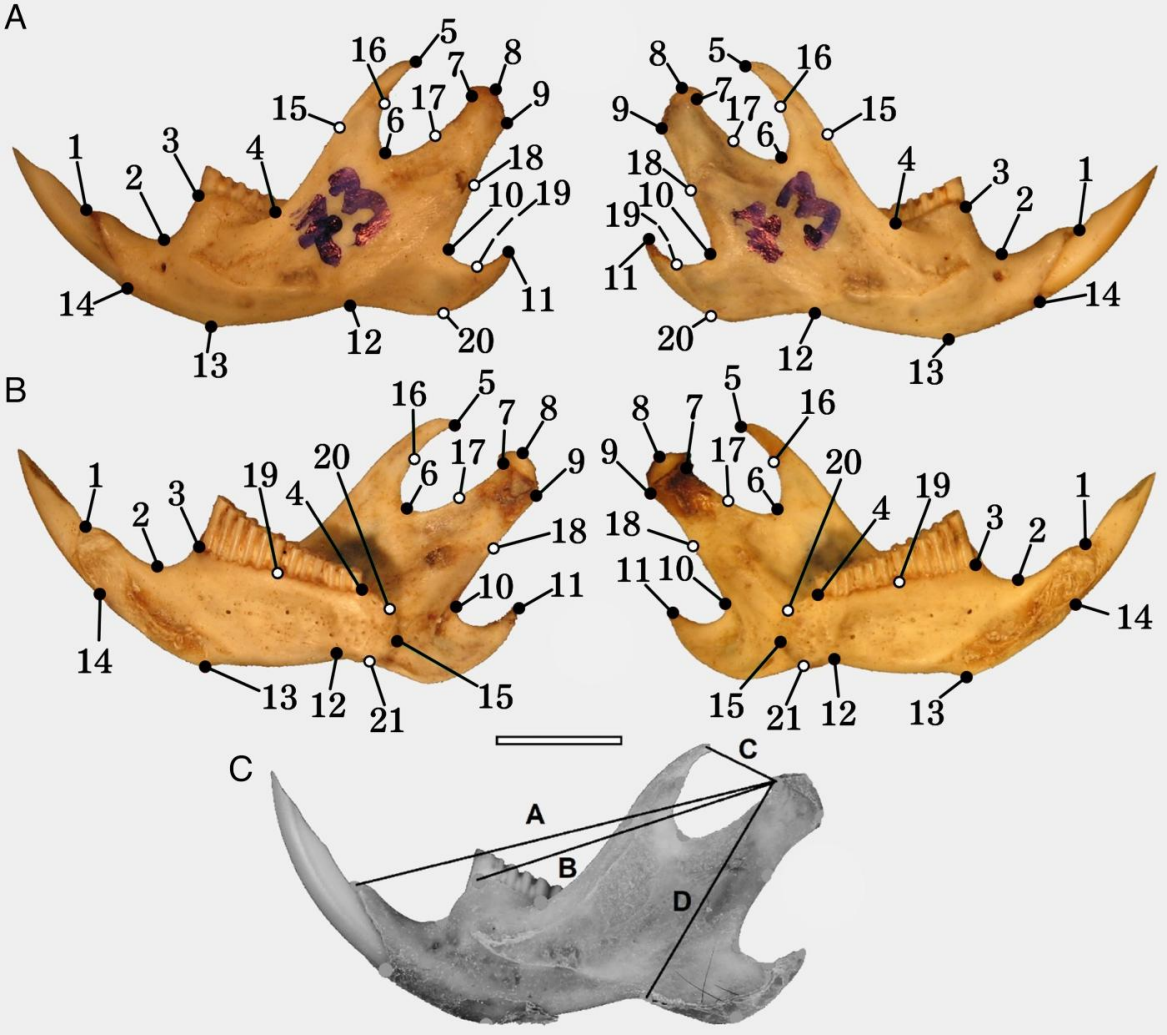
Locations (A: lateral projections of the left and right hemimandible respectively, B: medial projections of the right and left hemimandible respectively) of the anatomical landmarks (black dots) and the semilandmarks (white dots with black outline) and (C) four linear distances (A: incisor; B: molar; C: temporal; D: masseter) expressed as morphoecological indices. Numbers indicate the landmark numbers. *Microtus hartingi* hemimandibles are used as a reference. Scale bar: 5 mm.

Twenty landmarks from the labial and 21 from the lingual side of the mandible, including 6 mathematical landmarks for each side, were digitized (Fig. 1. and see Supplementary Figure 1 for the correspondence of landmarks) and curve lines representing the contour of a specimen were digitally drawn using the tpsDig32 software (Rohlf 2016). Data on the labial and lingual mandibles were separated and treated as 2 different datasets. In the case of *HarR*, some specimens were excluded from the analysis owing to too many missing landmarks. For each taxon, the mandibles were separated to 4 smaller datasets by: hemimandible side (left or right) and view (labial or lingual). Deformation degrees were treated as variables (individuals or factors).

### Statistical analysis

Descriptive statistics were obtained in Microsoft Excel 2010. Before geometric morphometric analysis the data were maintained using the R (R Core Team 2024) package geomorph (version 4.0.8) (Baken et al. 2021; Adams et al. 2024).

TPS files with landmarks and photo scale were uploaded into R. Then, missing landmarks were replaced by pseudo-data generated using the R *Estimate Missing* function from the geomorph package (Adams et al. 2024), using TPS estimation method. In total, 12.82% of the dataset landmarks were missing and were replaced with pseudo-data using the *Estimate Missing* (geomorph) function. Outliers were determined using the *plot Outliers* (geomorph) function (Adams et al. 2024). Outliers were excluded manually from the initial TPS file, and again uploaded to the R followed by missing landmark estimation. Received data were written to a new TPS file for the ongoing analysis. Curve landmark coordinates were also normalized in R using *warpRefOutline* (geomorph) function (Adams et al. 2024) and the received landmark coordinates were written into a .txt curve file for further use in the analysis.

To describe shape variation of the mandible among vole individuals of different deformation degrees, MorphoJ (Klingenberg 2011) and R were used. To calculate the influence of deformation degree on shape differences in mandible data, Procrustes ANOVA analysis was carried out. In addition, principal component analysis (PCA) and canonical variance (CVA) analysis were performed. PCA was used to summarize the overall shape variance across all specimens regardless of the sample group. CVA was applied to visualize differences among sample groups: N versus D for *HarR*, and N versus ID versus D for *Ank*.

Linear distances measurements were obtained using normalized centroid distances between landmarks (LM 5–7, LM 1–7, LM 7–12, LM 3–7) (Fig. 1A). The Euclidean distance formula was used for the calculations. All of the calculations were performed in R using TPS files with LM data. A number of studies have shown that mandibular morphoecological indices obtained using 4 characteristic measurements make it possible to investigate mandibular configurations that characterize the morphological functional features associated with differences in food processing mechanics in rodents (Hiiemae 1971; Anderson et al. 2014). This approach allows for quantifying trophic specialization in species and populations. Four morphoecological indices were calculated (Zorenko et al. 2023): temporal incisive = C/A, temporal molar = C/B, masseter incisor (MI) = D/A, and masseter molar (MM) = D/B (Fig. 1C). These indices served as indirect ecological criteria for characterizing interspecific or intraspecific differentiation (Gorodilova and Vasilyeva 2014).

The Mann-Whitney U test, implemented in R (function *wilcox.test*) was applied to assess differences in incisor length, differences in landmark distances between the groups of different deformation degrees, upper diastema length and in morphoecological indices values between the sample groups: N, ID (for tooth only), and D in *HarR*; and N, ID, and D in *Ank*. Group was used as the factor, and incisor length as the tested parameter.

Two-Sample Student's test implemented in R (function *t.test*) was performed to assess the impact of sex on the heterochrony manifestation, where sex was used as the factor, and age as the tested parameter.

## Results

### Sex and age

Impaired incisor growth was noted in both vole colonies: in 15.4% of *HarR* individuals (over 6 years, 222/1438) and in 10.3% of *Ank* (over 8 years, 170/1643). The impact of sex as a factor in the manifestation of heterochrony was not significant “as the proportion of males and females with deformations was almost equal in both populations.” In the *HarR* population, the overgrowth of the maxillary teeth was noted in 102 males and 120 females (only 8.2% more females). In the *Ank* population, 86 males and 85 females were affected. However, in both populations, heterochrony appeared at an earlier age in males compared with females: *Ank*—3.7 ± 0.17 and 4.1 ± 0.197 (Student's test, *t* = 1.5, *P* = 0.1) and *Har R*—3.8 ± 0.16 and 4.0 ± 0.16 months (Student's test, *t* = 0.91, *P* > 0.1), respectively, although the differences were not statistically significant. In both populations, rupture of the alveolar wall under M2 pressure was observed with higher prevalence in males than in females: 1.3× more often in *HarR* (29/35 N, 83% and 17/26 D, 65%), and 2× in *Ank* (9/21, 43%, and 3/14, 21%, respectively).

### Incisor growth

**Figure 2:**
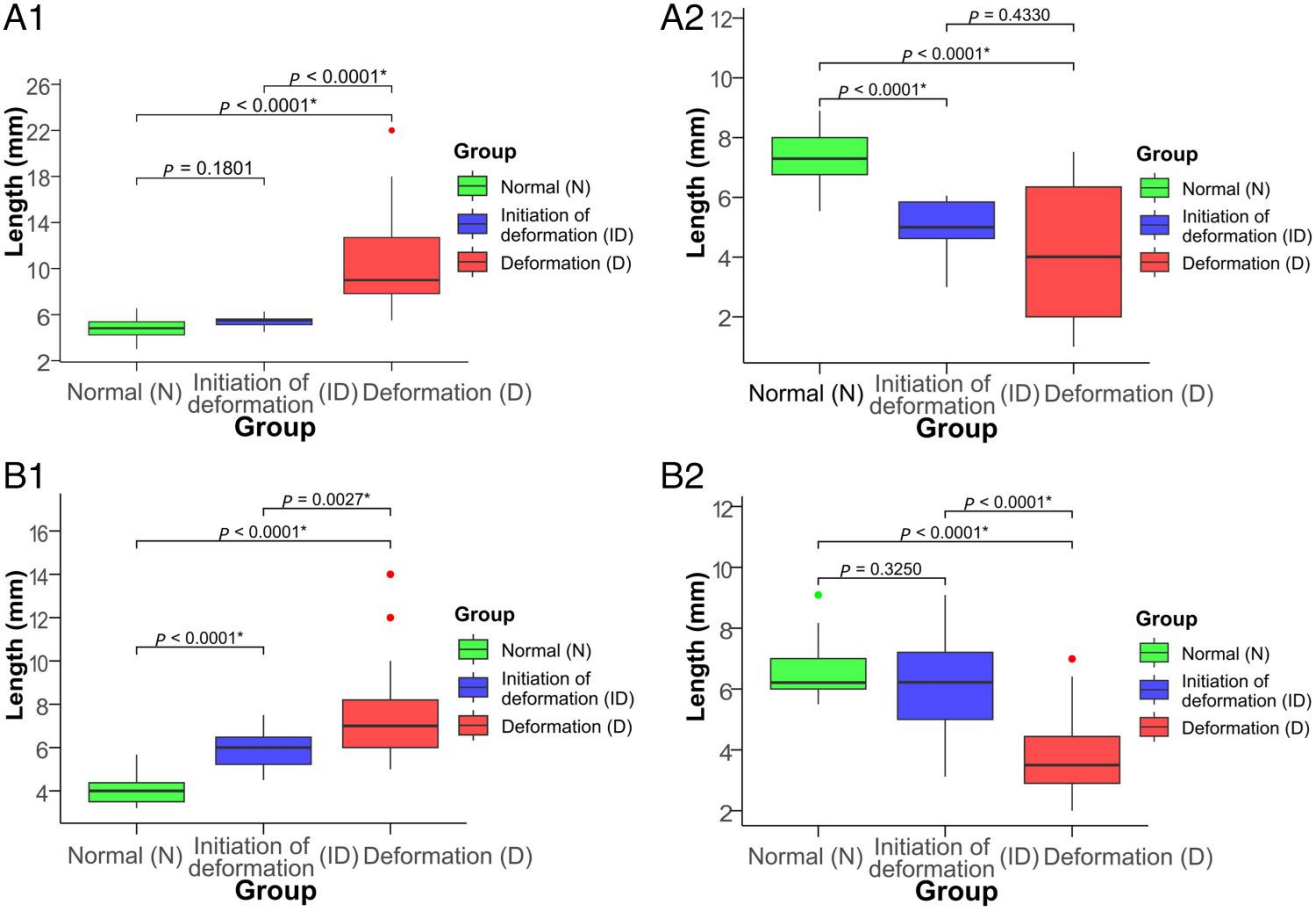
Incisor length changes at different stages of the deformation in two vole populations: *Microtus hartingi*: A1—maxillary incisors, A2—mandibular incisors; *Microtus hartingi ankaraensis*: B1– maxillary incisor, B2—mandibular incisor (N: normal, ID: initiation of deformation, D: deformation).

In healthy animals (N), the maxillary incisors are shorter than the mandibular incisors. However, in some voles, shortening of the lower and overgrowth of the upper incisors were recorded (ID, D). The initial stage (ID) of disruption in incisor growth began when the lengths of the maxillary and mandibular incisors became equal (Fig. 2).

In *HarR*, when incisor growth was impaired, the upper incisors elongated 2.3× compared with the norm (up to 18 mm), whereas the lower incisors shortened 1.8× (down to 1 mm). The increase in maxillary incisor length occurred faster than in mandibular incisors, sometimes with bending inward into the oral cavity or to the sides. A similar tendency was noted in *Ank*. However, fracture of the visible part of one or both mandibular incisors was more frequent (18/33, 54.5%) compared with *HarR*, where it occurred in only 9% (3/32) of voles. The difference in incisor length in *HarR* and *Ank* is shown in Fig. 2.

### Molar teeth

The molar teeth of *HarR* had a normal crown length. However, M2 (and less frequently M3) often ruptured the alveolar roof, noted both in N (51 of 68; 75%) and D (33 of 52; 64%) groups. The formation of gaps between m1–m2 and m2–m3 was observed. It was associated with chips or abrasion on the m2 surface from one or both sides of the tooth. In the N group, such changes were observed relatively rarely, occurring in 1.5% of mandibles and 7.4% of maxillae, whereas in the D group, the formation of gaps between molars was recorded in 26.9% of mandibles and 15.4% of maxillae. The length of M1–3 and m1–3 did not differ between the N and D groups (*P* > 0.05). However, in the D group, the diastema length decreased significantly (*P* < 0.001). In *Ank*, molars ruptured the alveolar roof less frequently than in *HarR*: 27.1% (19 of 70) of cases in the maxilla and 12.9% (28 of 70) in the mandible. On the contrary, gaps between molars were observed more frequently: in 42.8% of the maxillae and in 37.1% of the mandibles. The length of M1–3 and m1–3, as well as the diastema length, decreased in the ID group, especially in the D group compared with the N group.

### Linear morphometrics

No dentoalveolar system deformations were observed in the N group of *Ank* and *HarR*. In the D group, both populations showed similar changes: bone thickening in the mandibular corpus and ramus (posterior to the mandibular angle). Coronoid process shortening and articular process thinning were noted in some cases. Often, the deformations were more pronounced on one side than on the other. In the *HarR* D group, deformations were recorded more frequently on the left hemimandible (19.2%) than on the right (7.2%). Simultaneous changes in both hemimandibles were noted in 19.2% of cases. In the *Ank* population, at theID, mandibular deformations were slightly pronounced and occurred either on the left side (8.3%) or on both sides (11.1%). Later, in the D group, the right hemimandible was affected more often (35.3%) than the left (12%). Slight deformation of both hemimandibles was rarely observed (6%). In the living animal, mandibular deformation usually was not notable. In HarR, only 4 out of 122 (3.3%), and in *Ank*, 3 out of 70 (4.3%) living voles had externally visible changes.

The distance means and standard deviations for left hemimandibles are presented in Table 2. These distances were used to calculate mandibular morphoecological indices. The significance of the differences in morphoecological indices between the groups of both vole populations is presented in Table 3. In *HarR*, only the difference between LM 1–7 changed significantly, but in *Ank* the distance between LM 1–7 for N versus D and between LM 3–7 for ID versus D groups.

**Table 2:** Means and standard deviations (SE) of 4 linear centroid distances between landmarks (mm) of the left hemimandibles of 2 vole populations.

Population	Traits			
	LM 5–7^a^ mm ± SE	LM 1–7^a^ mm ± SE	LM 7–12^a^ mm ± SE	LM 3–7^a^ mm ± SE
	Left	Left	Left	Left
*Ank* normal (N)	2.66 ± 0.27	16.24 ± 0.53	9.33 ± 0.36	12.12 ± 0.44
*Ank* initiation of deformation (ID)	2.78 ± 0.29	16.11 ± 0.91	9.54 ± 0.68	11.87 ± 0.76
*Ank* deformation (D)	2.87 ± 0.28	15.29 ± 0.85	9.22 ± 0.58	11.39 ± 0.80
*HarR normal (N)*	3.26 ± 0.41	17.04 ± 0.83	10.13 ± 0.44	12.65 ± 0.64
*HarR* deformation (D)	3.10 ± 0.39	17.47 ± 3.62	10.14 ± 0.50	12.58 ± 0.65

*HarR*, *Microtus hartingi* from the Rhodopes; *Ank*, *Microtus h. ankaraensis*; N, normal; ID, initiation of deformation; D, deformation.

^a^See the corresponding landmarks in Fig. 1.

**Table 3:** Mann–Whitney *U* test results comparing the distances between the left hemimandibles with different deformation degree of 2 vole populations.

Distance	Group *P* values			
	N vs D		N vs ID	ID vs D
	*Ank*	*HarR*	*Ank*	*Ank*
LM 1–7	**0**.**001**	**0**.**038**	0.811	**0**.**005**
LM 5–7	0.160	0.271	0.2	0.16
LM 7–12	0.584	0.848	0.077	0.137
LM 3–7	**0**.**004**	0.326	0.472	0.057

*HarR*, *Microtus hartingi* from the Rhodopes; *Ank*, *Microtus h. ankaraensis*; N, normal; ID, initiation of deformation; D, deformation; boldfaced, a significant difference.

The analysis of morphoecological indices revealed no significant difference between the ID versus D group in *Ank*. In *HarR* the only significant difference observed was in the MI index (*P* = 0.021). However, comparing the N versus D and N versus ID *Ank* groups, the significant difference was observed between all indices (*P* = 0.038 – < 0.001). No significant differences were observed between the right hemimandibles in any of the calculations (*P* > 0.05), so the data are not presented.

### Geometric morphometrics

**Figure 3:**
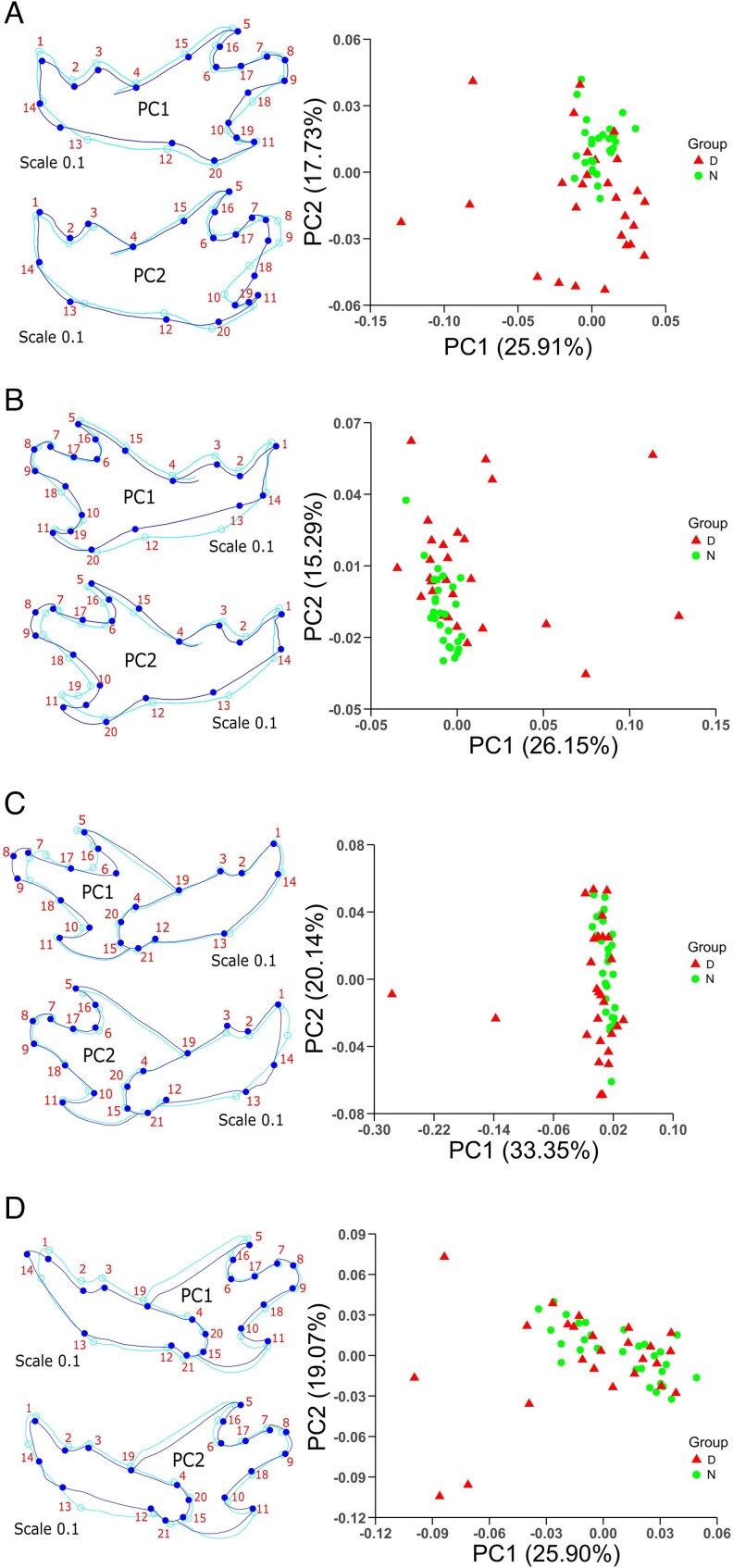
Scatter plots of the PCA (right) and positive directional shape changes of PC1 and PC2 (left) of the *Microtus hartingi* N and D datasets: A) labial left, B) labial right, C) lingual left, D) lingual right mandibles. Circles—normal (N); triangles—deformed (D) mandibles. Blue dots indicate landmarks after shape deformation; red numbers indicate landmark numbers; the scale factor for deformation visualization is 0.1. The outline shows deformation of the mandibles by PCs, from negative (dark-blue) to positive (light-blue) sides of the axes, and vice-versa on Figure A for PC2, and Figure C (N: normal; D: deformation).

**Figure 4:**
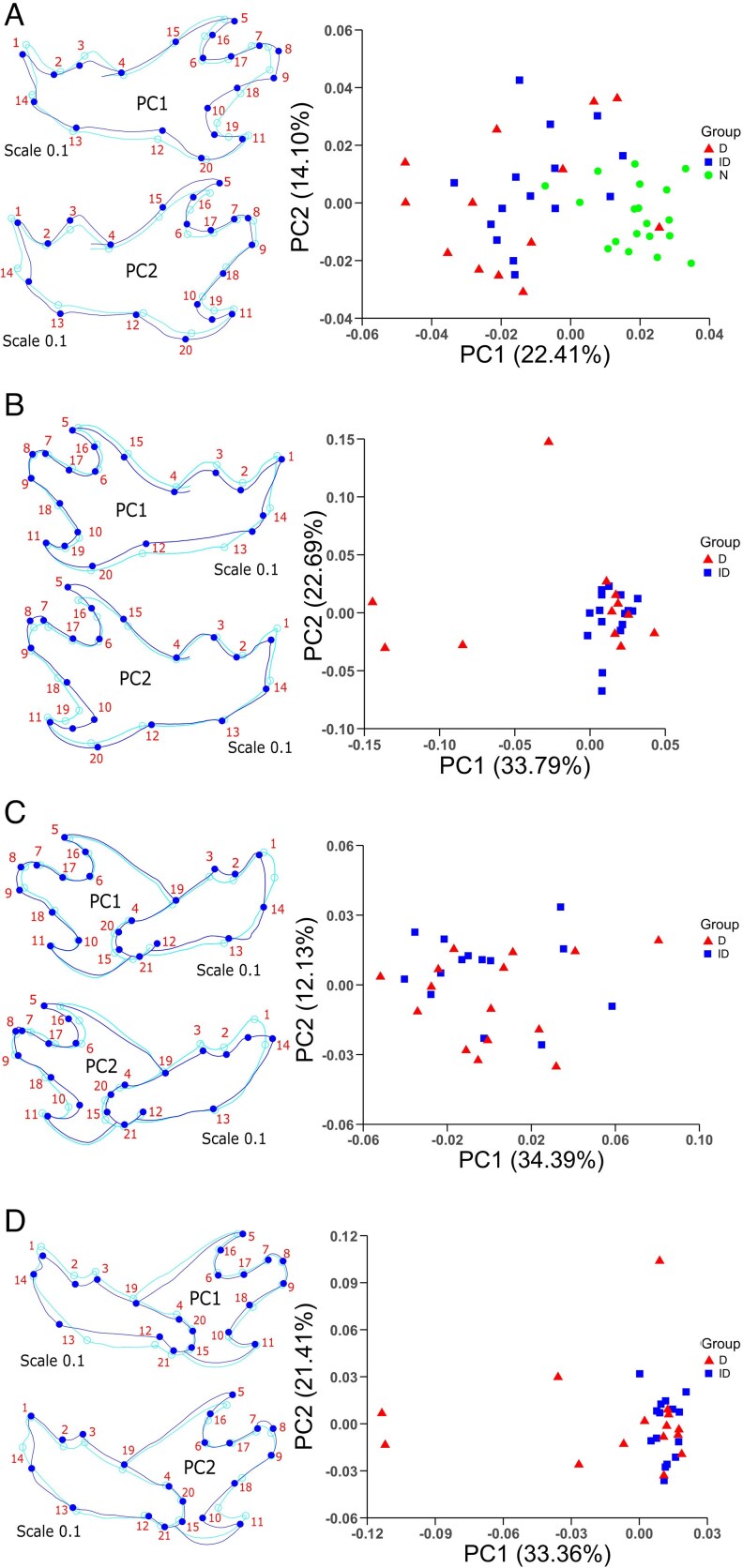
Scatter plots of the PCA (on the right) and positive directional shape changes of PC1 and PC2 (on the left) of the D and N datasets of the *Microtus hartingi ankaraensis* of A) labial left, B) labial right, C) lingual left, D) lingual right mandibles. Dots—normal (N), Squares—initiation of deformation (ID); triangles—deformed (D) mandibles. Blue dots on the shape changes plots indicate landmarks after shape deformation; red numbers indicate landmark numbers; The outline shows deformation of the mandibles by PCs, from negative (dark-blue) to positive (light-blue) sides of the axes, and vice-versa on Figure D for PC2. (N: normal; ID: initiation of deformation; D: deformation).

Both *HarR* and *Ank* showed similar deformation “direction” patterns with significant differences between groups of different deformation degrees (N, ID, D) (Figs. 3 and 4; Supplementary Figure 2 and 3). Procrustes ANOVA revealed significant difference in shape among all sample groups (*P* < 0.001) except for the lingual left *Ank* hemimandibles (*P* = 0.502), whereas centroid size (CS) differences were not significant in most comparisons (*P* > 0.05), except for the labial left *Ank* hemimandible (*P* = 0.027). All data are presented in Tables 4 and 5.

**Table 4:** Procrustes ANOVA results of *Microtus hartingi* comparing normal and deformed hemimandibles.

Projection	side		F factor	*P* value
Labial	Left	Centroid size	1.93	0.171
		Shape	3.79	**<0**.**001**
	Right	Centroid size	0.09	0.762
		Shape	3.07	**<0**.**001**
Lingual	Left	Centroid size	0.69	0.410
		Shape	3.43	**<0**.**001**
	Right	Centroid size	0.66	0.422
		Shape	2.82	**<0**.**001**

F, Goodal's F; CS, centroid size; boldfaced, a significant difference.

**Table 5:** Procrustes ANOVA results of *Microtus hartingi ankaraensis* comparing normal, initiation of deformation, and deformed hemimandibles for labial left.

Projection	side		F factor	*P* value
Labial	Left	Centroid size	3.92	**0**.**027**
		Shape	4.63	**<0**.**001**
	Right	Centroid size	2.20	0.150
		Shape	2.17	**0**.**001**
Lingual	Left	Centroid size	2.67	0.114
		Shape	0.98	0.502
	Right	Centroid size	2.19	0.150
		Shape	1.96	**0**.**001**

F, Goodal's F; CS, centroid size; boldfaced, a significant difference.

The PCA and outline shape changes graphs (Figs. 3 and 4) demonstrate deformation patterns in both vole populations, supporting the Procrustes ANOVA results: the mandible corpus and the coronoid process extension, raised molar alveolus, deformation of the incisor alveolus, shortening of the diastema (LM 1–5, 15, 12–14, 20), and narrowing of spaces between the coronoid and articular, the articular and angular processes, and extension of the condyloid process in deformed jaws (LM 6–11, 16–19); however, the space between the coronoid and condyloid processes is less affected and may show slight extension in some cases. From the lingual side, widening and enlargement of the m3 alveolus (LM 12, 21, 15, 20, and 4) was noted. Deformation patterns described by other landmarks are similar to the labial side of the mandible: extension of the mandible corpus, coronoid process and diastema (LM 1–3, 5, 13, and 14), extension of the condylar process with narrowing of spaces between the coronoid and articular, articular and angular processes (LM 5–11, 17–19).

When photographing the mandible, it was noted that in deformed mandibles the m3 alveolus was often broken and/or crushed and significantly thinned. However, it was also observed in normal mandibles but with lower occurrence. Also, the incisor alveolus of the ID and D mandibles was usually deformed. The coronoid process sometimes was damaged or absent in both normal and deformed bones; however, this was more common in the D group.

The first 2 PC components were used to describe the shape changes in each sample group. PC1 and PC2 components together explained from 34.39% to 58.48% of the variance across all groups, representing the lowest and highest explained variance, respectively. Fig. 2A1, A2, B1, B2 shows that normal and deformed mandibles in *HarR* form mostly distinct, easily observable groups with overlapping patterns. It can be seen that the D groups of every mandible set have higher spread on the PCA scatter plots, which means higher variation in shapes. In addition, the N groups are located more positively relative to the D groups along the PC1 and PC2 axes, with the exception of PC2 of the labial left hemimandibles (Fig. 2A), explained by the PC2 parameter, where the D jaws are slightly more positive than the N hemimandibles (Fig. 2B, C, D). This is also supported by PCs outline graphs for every sample group. For the *Ank*, PCA scatter plots and deformation plots are presented on a Fig. 3A, B, C, D. Fig. 3A displays the PCA analysis results for the labial left mandibles. This sample group, unlike the rest for *Ank*, also contains the N mandibles group. The scatter plot on the right clearly displays distribution of 3 mandible groups, where on the PC1, the N mandibles are located more positively on the axis than ID, and D, and have lower dispersion, intersecting only with the ID sample group, except one D mandible, which point can be observed right in the middle of the N group points. Group D has the most negative values, intersecting with ID on the positive side. Groups D and ID have higher variance than the N group, which means there is higher shape variability compared with N. The level of deformation presented in PC2 is very low.

The outline of *Ank* mandibles showed a deformation pattern similar to the *HarR*. On the Fig. 3B, the ID group has lower dispersed and slightly more negative values on both PC1 and PC2 axes. It can be noted that on a scatter plot of the Fig. 3C, both ID and N samples have similar dispersion, except the ID group having slightly more positive values by the PC2 axis. This was also confirmed by the Procrustes ANOVA results, where both CS and shape have *P* value >0.05. Fig. 3D has a similar pattern to Fig. 3B, however, on the PC1 axis; the D group has more negative values than ID.

**Figure 5:**
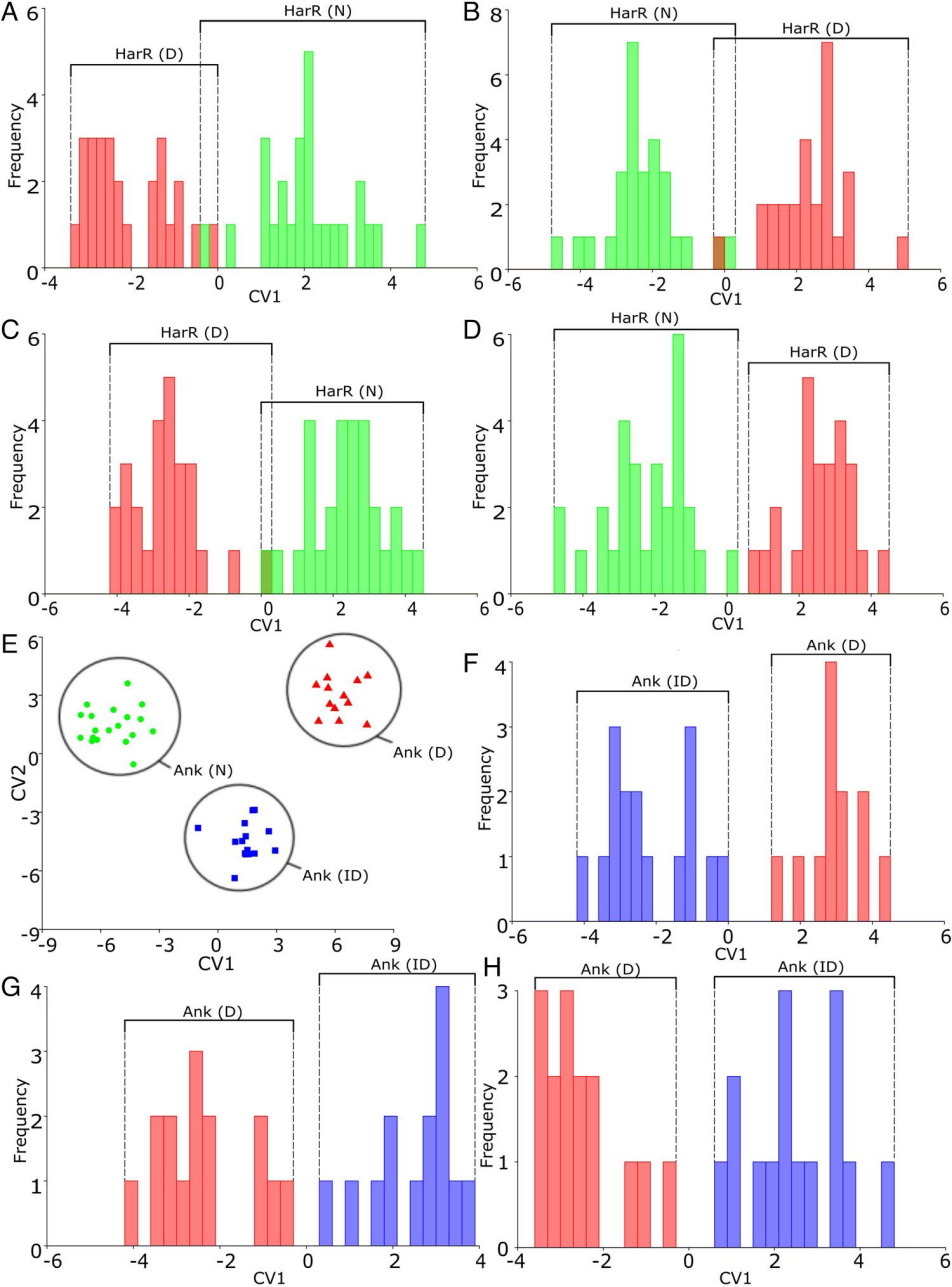
CVA analysis histogram (A, B, C, D, F, G and H) and scatter (E) plots for *Microtus hartingi* from Rhodopes A) labial left, B) labial right, C) lingual left, D) lingual right hemimandibles (N and D datasets); and *Microtus hartingi ankaraensis* E) labial left, F) labial right, G) lingual left, H) lingual right mandibles (N, ID and D datasets). (N: normal, ID: initiation of deformation, D: deformation).

For the CVA results, because each comparison has only 2 sample groups, CV1 explained 100% of the variability, except for the labial left hemimandibles of *Ank*, where 3 groups are present. In this case, CV1 explained 70.16% of the variance, whereas CV2 accounted for 29.84% (Fig. 5E). CVA revealed significant shape differences between the *HarR* N and D groups, except for the labial left mandibles, where the Mahalanobis distance (*P* = 0.085) and Pillai's trace (*P* = 0.085) were not significant (Supplementary Table 1). For *Ank*, the Mahalanobis distance was not significant in all groups (*P* > 0.05). Procrustes distance *P* values were significantly different in all sample groups (*P* < 0.05) except for the lingual left mandibles (*P* = 0.410). Permutation test Goodal's F showed the same significance pattern. Pillai's trace was statistically significant only for the labial left samples (*P* < 0.001) (Supplementary Table 2).

The CVA histograms showed very low (Fig. 5A, B, C) or no overlapping among groups with different deformation degrees (Fig. 5D, F, G, H). The CVA scatter plot (Fig. 5E) showed no overlapping along the PC1 axis, but there was some overlap between the N and D groups on the PC2 axis. The CVA outline deformation plots (Supplementary Figure 2 and 3) showed a similar deformation pattern to the PCA (Figs. 3 and 4). However, the narrowing of spaces between the coronoid and condylar, and the condylar and angular processes is better observed on CVA plots and is more pronounced compared with the PCA plots.

## Discussion

Conducted research demonstrates a high plasticity of the Harting's vole dentoalveolar system. Three morphotypes were identified in Harting's vole laboratory colonies: 1) *maxillary incisor elongation*, sometimes with bending inward into the oral cavity or to the sides; 2) *molar elongation*, with maxillary or mandibular bone perforation by molar apexes in some cases; 3) *mandibular bone shape deformations*. It was also noted that mandibular shape changes are always asymmetrical, which is especially pronounced in the later stages of the condition. One hemimandible is always more deformed, than the other. Asymmetrical morphotypes of molar teeth were identified in different voles species (Kovaleva et al. 2021) All malformations are predictably determined by the complex modular structure, functioning, and high sensitivity of the rodents' dentoalveolar system to environment changes. Various environmental factors, including stress, may lead to developmental anomalies in the rodents' ever-growing teeth at both individual, population and species levels (Ginot et al. 2018; Parés-Casanova 2019; Kovaleva et al. 2021). Particular attention is paid to attempt to find a relationship between morphotypic and genetic (Klingenberg and Leamy 2001) as well as morphological and molecular-genetic variation of individuals and species (Kovaleva et al. 2019), through the analysis of corresponding distance matrices. A high correlation was found among these 3 types of variation. Two hypotheses have been proposed to explain the observed correlation: random mutation and directional selection (Hershberg and Petrov 2008). PCA analysis as well as CVA analysis revealed similar and unidirectional changes in mandibles in both Harting's vole populations (Figs. 3 and 4; Supplementary Figure 2 and 3), namely, dorsoventral extension of the mandibular corpus and the region of the mandibular processes with a narrowing of notches between them. It is possible to assume the presence of a selective mechanism responsible for the observed shape changes. Further research is needed to identify the correlation between molecular-genetic variation and changes in mandibular shape.

On average, 15.4% of *HarR* individuals exhibited a maxillary incisor elongation. Prevalence remained the same in F2–F6 with slight variation, indicating a hereditary nature of this trait. The impact of genetic factors was mentioned in various studies (Gill and Bolles 1982; Ida-Yonemochi et al. 2002; Imbschweiler et al. 2011). Jheon et al. 2015 suggested that a spontaneous mutation may cause misregulation in the growth of hypselodont teeth, herewith, incisor and molar overgrowth may result from different mutations. Our findings regarding mandible and teeth deformation are consistent with the concept of modularity in these morphological structures (Klingenberg 2003; 2009; Renaud et al. 2012; Klingenberg and Marugán-Lobón 2013). Thus one animal may exhibit both maxillary incisor and apical molar elongation, but these traits may not necessarily be related. Seemingly, anomalies in growth might appear independently, as noted by Jheon et al. (2015), but further research is needed. In the *Ank* population, incisor heterochrony did not develop in the first 10 laboratory generations. However, after the exposure to physical and social stress (Zorenko 2022), the first individuals with impaired incisor growth appeared. This allowed us to hypothesize that stress could be a stimulus for the onset of incisor growth anomalies due to increased sensitivity to stress. In this population, more than 8 years of laboratory keeping, the manifestation of heterochrony was observed on average in 10.3% of the animals.

We assume that dentoalveolar system deformations may be the result of a synergistic interaction between genetic and epigenetic factors. In our previous study (Kaija et al. 2024) it was hypothesized that the incisor overgrowth could be induced by epigenetic changes due to experimental stress exposure, which resulted in the disruption of the synchronous restoration of incisor length, leading to increased maxillary and decreased mandibular incisor growth speed. On the proximal end of the maxillary incisor (growth zone), several layers of stem cells, regulated by a complex gene network, responsible for dental growth, symmetry and proportional length of teeth are located (Wang et al. 2007; Vogel et al. 2015). So, as noted by Jheon et al. (2015), a mutation in stem cells could lead to the deformation onset. Typically, maxillary incisor growth speed in different vole species ranges from 0.9 to 1.4 mm per week, whereas the molar growth speed is slightly slower—from 0.4 to 1.2 mm per week (Golenishchev and Koenigswald 1978). Trimming of overgrown incisors, commonly used in veterinary practice (Capello 2008) was not effective, as the incisor length was restored within 7–10 days. The incisor overgrowth we examined can be determined as a pathology, which inevitably leads to the animals' death.

In Harting's vole, the incisor overgrowth was usually developed in animals aged 4–6 months. Only a few animals exhibited the initiation of this condition at an earlier age (2.5–3 months) or at a later age (8–10 months). Voles could produce 1–5 L before the initiation of overgrowth. Many authors note that these changes are age related. For example, in Dontas et al. 2010 study of aging in Wistar rats, not a single case of the incisor overgrowth was observed during the first year of life. However, by the 3rd year, 32.5% of the animals exhibited incisor overgrowth (Dontas et al. 2010). In *HarR*, the low prevalence rate of this morphotype in each generation and its late expression might indicate that over the course of long evolution this negative trait in the population has been limited but the morphotype is not completely eliminated. On the contrary, in *Ank* the proportion of the overgrown incisor morphotype noticeably decreased by the fourth generation (unpublished data). Jheon et al. 2015 noted the reduction in molar apical elongation as early as in the F2 generation. In nature, the *HarR* population exists in the highly stressful environment of the Rhodope foothills, characterized by habitat fragmentation, a lack of new suitable settlement sites, a higher risk of inbreeding, and, in recent years, growing anthropogenic pressure. In contrast, the *Ank* population exists in the steppes of Central Anatolia. This habitat is more suitable for voles, so the animals are less affected by the environmental stress. This suggests that this difference in 2 Harting's vole populations is explained by their different life histories: in *HarR*, the morphotype could have become established at a stable level as a result of the ecological stress initiated epigenetic rearrangement over the course of many millennia (Whitlock 2003). In contrast, in *Ank*, exposure to the experimental stress caused a temporary increase in the morphotypic diversity, but the trait was then “washed out” in the absence of persistent stress exposure.

Hypothetically, incisor malocclusion could be the reason for the maxillary incisor overgrowth. However, in Harting's vole we noted simultaneous growth impairment of both maxillary and mandibular incisor. We have not observed a single case of the mandibular incisor overgrowth, although this is possible in some vole species (noted in *M. arvalis* Jentzsch et al. 2020). Besides, anterior malocclusion occurs at an early age and is often accompanied by decreased food intake, emaciation and ptyalism in some cases (Capello 2008), which was not observed in our case. Although voles could exhibit a slight shift of the tooth rows relative to each other, resulting in the maxillary molars diverging from the mandibular molars (right or left malocclusion). Asymmetry of the deformed hemimandibles was also observed in Harting's vole. It was noted that in *HarR*, the left hemimandible is deformed more often, but in *Ank*—the right hemimandible. Changes in the mandible and dentition, such as the non-functioning M2–M3 and/or m3, or disruption of occlusal contacts between the dentition and incisors, can alter the coordinated action of the masticatory muscles. This may cause the animal to change the normal mandible position while chewing to make it more comfortable and less painful. As our data showed, there was a high positive correlation between incisor overgrowth and mandible deformation. This was also noted by other authors (Baskin et al. 2021). We believe that molar malocclusion with a consequent hemimandible displacement is what prompts the incisor elongation. These data are supported by a Baskin et al. (2021) study, where the artificial defect formation in the area of the mandibular ramus resulted in incisor symmetry disruption in rats immediately after the surgery. However, as healing progressed, not only the mandibular bone was restored, but also the symmetry of the incisors was reestablished. This highlights the high dentoalveolar system plasticity and an interconnection between its components.

The molar elongation morphotype was expressed differently. Molars could exhibit both coronal and, more often, apical elongation. Overgrowth of the tooth apex results in stretching of the alveolar bone with possible perforation and formation of protuberances in some cases. We noted that molar apical elongation did not affect animal welfare, although in up to 75% of the N skulls obtained from the *HarR* wild and laboratory population we noted M2/M3 apical elongation with perforation of the alveolar bone of the skull. Perforation of the m3 alveolus was noted less frequently. According to Gill and Bolles (1982), California voles *M. californicus* lived up to 15 months despite the deformation of the molar teeth. However, a number of other authors noted the death of animals with molar apical elongation due to the formation of the large alveolar bone protuberances inside the cranial cavity. Clinical traits such as ocular discharge, abnormal mastication, dyspnea, abnormal mentation and weight loss were noted (Imbschweiler et al. 2011; Imai et al. 2018), but these symptoms were not observed in our case. Using computed microtomography, it was demonstrated that *M. hartingi* with normal molar crown length may exhibit M2–M3 and m3 alveolar bone protrusion (Golenishchev et al. 2022). This was similar to what we observed in our study.

In both Harting's vole populations, gaps between the first and second molars, and sometimes between the second and third molars were formed both in the mandible and in the maxilla. In *HarR*, molar teeth deformations were observed in 42% of the animals, whereas in *Ank*, the deformation rate was as high as 71%. One of the reasons may be chipping or resorption of the tooth prism, as well as deformation and expansion of the periodontal fissure, which could also be a consequence of a mineral metabolism violation. Bone formation and remodeling are known to be a part of calcium and phosphate homeostasis (Song 2017). Misregulation of this process may result from the stress impact. Thinned bones can easily break when chewing (Golenishchev et al. 2022). Alveolar bone deformation in *Ank* occurred less frequently than in *HarR*. A possible explanation is that the phosphorus and calcium metabolism disturbance in *HarR* has been fixed in the population over a long time, whereas in *Ank*, the phosphorus-calcium metabolism has changed in several years of laboratory keeping.

Outside of rare cases, mandibular deformation was not observable in the living vole. Only 3–4% of the animals exhibited visible compactions (odontomas with calcareous deposits), localized in the region of the mandibular corpus. Both PCA and CVA GM analyses revealed similar mandibular deformation patterns in D groups of mandibles in both vole populations (Figs. 3 and 4; Supplementary Figure 2 and 3). It is important to mention that CVA results should be treated with caution, as these results might be affected by the artificial separation by groups (Mitteroecker and Bookstein 2011). Main patterns were the enlargement of the mandibular corpus and ramus, the diastema shortening, enlargement of the m3 alveolus and incisor alveolus deformation. The notches between coronoid and articular, articular and angular processes narrowed, whereas the expansion of the condyloid process in deformed jaws occurred. However, space between the coronoid and articular processes is less affected and in some cases may widen slightly. Shortened diastema, changes in the shape and size of the mandibular processes, to which adductor muscles are attached, suggest alterations in the biomechanics of the mandible.

The articular process is the most significant part of the mandibular apparatus (Nikol'sky 1997) and all of the morphoecological indices are calculated relative to it. They show a correlation between dietary specialization and mandibular structure and reflect the biomechanics of the jaws. In *HarR*, despite the pronounced differences in mandibular distances and the shape of the N and D groups, only the MI index changed significantly. This index reflects the relationship between 2 distances: from the articular process (LM7) to the most concave part of the mandibular corpus (LM12) and from the articular process (LM7) to the upper border of the incisor alveolus (LM1) (Fig. 1). Both of these distances were slightly decreased in the deformed mandibles—by 0.18 and 0.20 mm, respectively. This resulted in a slight MI index increase. Regardless of the mandibular deformation, chewing in *HarR* voles was not impaired. A different picture was observed in the *Ank* population. Both at the initial and later stages of the mandibular deformation (ID and D), the voles apparently encountered feeding difficulties, which led to changes in all morphoecological indices compared with normal (N) mandibles (Fig. 3A). Most likely, animals process the food always using only the less deformed hemimandible, whereas the second hemimandible serves as a balance.

In conclusion, we can note that despite the presence of the discovered dentoalveolar system deformation morphotypes (apical molar elongation, coronal incisor elongation, and mandibular deformation), the animals were able to survive for a long time and produce offspring. It can be assumed that the incisor length change may affect the mandibular bone remodeling process, causing the deformations we observed. These deformations might compensate for the incisor length changes. As a result, the dentoalveolar system biomechanics are preserved and the bite force is maintained on a sufficient level. This allowed animals to feed without significant disruptions.

## Supplementary Material

zoaf052_Supplementary_Data

## References

[zoaf052-B2] Adams D, Collyer M, Kaliontzopoulou A, Baken E. 2024. Geomorph: Software for geometric morphometric analyses. R package version 4.0.9. [cited 2025 January 17]. Available from: https://cran.r-project.org/package=geomorph.

[zoaf052-B3] Anderson PS, Renaud S, Rayfield EJ, 2014. Adaptive plasticity in the mouse mandible. BMC Evol Biol 14(1):1–9. 10.1186/1471-2148-14-85 PMC4002541 24742055

[zoaf052-B4] Baken E, Collyer M, Kaliontzopoulou A, 2021. Geomorph v4.0 and gmShiny: enhanced analytics and a new graphical interface for a comprehensive morphometric experience. Methods Ecol Evol 12(12):2355–2363.

[zoaf052-B5] Baskin JZ, White BM, Vasanji A, Love TE, Eppell SJ, 2021. Mandible biomechanics and continuously erupting teeth: a new defect model for studying load-bearing biomaterials. Biomedicines 9(7):730. 34202189 10.3390/biomedicines9070730 PMC8301467

[zoaf052-B6] Capello V , 2008. Diagnosis and treatment of dental disease in pet rodents. J Exot 17(2):114–123.

[zoaf052-B7] Dontas IA, Tsolakis AI, Khaldi L, Patra E, Lyritis GP, 2010. Malocclusion in aging Wistar rats. J Am Assoc Lab Anim Sci 49(1):22–26. 20122311 PMC2824962

[zoaf052-B8] Engström C, Kiliaridis S, Thilander B, 1986. The relationship between masticatory function and craniofacial morphology. II A histological study in the growing rat fed a soft diet. Eur J Orthod 8(4):271–279. 3466802 10.1093/ejo/8.4.271

[zoaf052-B9] Gill AE, Bolles K, 1982. A heritable tooth trait varying in two subspecies of *Microtus californicus* (Rodentia: Cricetidae). J Mammal 63(1):96–103.

[zoaf052-B10] Ginot S, Agret S, Claude J, 2018. Bite force performance, fluctuating asymmetry and antisymmetry in the mandible of inbred and outbred wild-derived strains of mice (*Mus musculus domesticus*). Evol Biol 45(3):287–302.

[zoaf052-B11] Goedendorp MM, Van der Werf SP, Bleijenberg G, Tummers M, Knoop H, 2013. Does neuropsychological test performance predict outcome of cognitive behavior therapy for Chronic Fatigue Syndrome and what is the role of underperformance? J Psychosom Res 75(3):242–248. 23972413 10.1016/j.jpsychores.2013.07.011

[zoaf052-B12] Golenishchev FN, Koenigswald B, 1978. Rate of growth of rootless teeth in Microtinae (Mammalia, Rodentia). In: Strelkov PP, editor. Functional morphology and systematics of mammals, Vol. 79. St. Petersburg: ZIN AN SSSR, 102–104.

[zoaf052-B13] Golenishchev FN, Zorenko TA, Petrova TV, Voyta LL, Kryuchkova LY et al, 2022. Evaluation of the “bottleneck” effect in an isolated population of *Microtus hartingi* (Rodentia, Arvicolinae) from the Eastern Rhodopes (Bulgaria) by methods of integrative analysis. Diversity (Basel) 14(9):709.

[zoaf052-B14] Gorodilova JV, Vasilyeva IA, 2014. [Geometric morphometry of the mandible in chromosomal races of the Ural harvest mouse (*Sylvaemus uralensis* Pallas, 1811): taxonomic and ecological aspects]. Uspehi Sovremennogo Estestvoznaniya 11:19–24. in Russian.

[zoaf052-B15] Grϋneberg H , 1963. The Pathology of Development. A Study of Inherited Skeletal Disorders in Animals Oxford: Blackwell Scientific Publications.

[zoaf052-B16] Gromov IM, Polyakov I, 1977. [Mammals, Voles Microtinae] Leningrad: Nauka. (in Russian).

[zoaf052-B17] Harvey SB, Alworth LC, Blas-Machado U, 2009. Molar malocclusions in pine voles (*Microtus pinetorum*). J Am Assoc Lab Anim Sci 48(4):412–415. 19653952 PMC2715934

[zoaf052-B18] He M, Wang P, Li B, Wang Y, Wang X, 2021. Rodent incisor and molar dental follicles show distinct characteristics in tooth eruption. Arch Oral Biol 126:105117. 33845260 10.1016/j.archoralbio.2021.105117

[zoaf052-B19] Hershberg R, Petrov DA, 2008. Selection on codon bias. Annu Rev Genet 42(1):287–299. 18983258 10.1146/annurev.genet.42.110807.091442

[zoaf052-B20] Hiiemae K , 1971. The structure and function of the jaw muscles in the rat (*Rattus norvegicus* L.) III. The mechanics of the muscles. Zool J Linn Soc 50(1):111–132.

[zoaf052-B21] Ida-Yonemochi H, Noda T, Shimokawa H, Saku T, 2002. Disturbed tooth eruption in osteopetrotic (op/op) mice: histopathogenesis of tooth malformation and odontomas. J Oral Pathol 31(6):361–373. 10.1034/j.1600-0714.2002.00087.x 12201247

[zoaf052-B22] Imai DM, Pesapane R, Conroy CJ, Alarcón CN, Allan N et al, 2018. Apical elongation of molar teeth in captive *Microtus* voles. Vet Pathol 55(4):572–583. 29665753 10.1177/0300985818758469

[zoaf052-B23] Imbschweiler I, Schauerte N, Henjes C, Fehr M, Baumgärtner W, 2011. Odontogenic dysplasia in the molar teeth of Steppe lemmings (*Lagurus lagurus*). Vet J 188(3):365–368. 20573534 10.1016/j.tvjl.2010.05.017

[zoaf052-B24] James MJ, Järvinen E, Wang XP, Thesleff I, 2006. Different roles of Runx2 during early neural crest–derived bone and tooth development. J Bone Miner Res 21(7):1034–1044. 16813524 10.1359/jbmr.060413

[zoaf052-B25] Jentzsch M, Kraft R, Lemkul A, Kapischke HJ, Maternowski HW et al, 2020. Anomalies and pathological changes of skulls and dentition of wild small mammal species from Germany. J Vertebr Biol 69(4):1–19.

[zoaf052-B26] Jheon AH, Prochazkova M, Sherman M, Manoli DS, Shah NM et al, 2015. Spontaneous emergence of overgrown molar teeth in a colony of Prairie voles (*Microtus ochrogaster*). Int J Oral Sci 7(1):23–26. 25634121 10.1038/ijos.2014.75 PMC4817538

[zoaf052-B27] Kaija LP, Zorenko T, Kagainis U. 2024. Heritable epigenetic effects of stress on occlusal disharmony in *Microtus hartingi* vole. In: Abstract book of the 82nd International Scientific Conference of the University of Latvia. Riga, Latvia, University of Latvia, 19.

[zoaf052-B28] Kaku M, Yamauchi M, 2014. Mechano-regulation of collagen biosynthesis in periodontal ligament. J Prosthodont Res 58(4):193–207. 25311991 10.1016/j.jpor.2014.08.003 PMC4253671

[zoaf052-B29] Klingenberg CP , 2003. Developmental integration in a complex morphological structure: how distinct are the modules in the mouse mandible? Evol Dev 5(5):522–531. 12950630 10.1046/j.1525-142x.2003.03057.x

[zoaf052-B30] Klingenberg CP , 2009. Morphometric integration and modularity in configurations of landmarks: tools for evaluating a priori hypotheses. Evol Dev 11(4):405–421. 19601974 10.1111/j.1525-142X.2009.00347.x PMC2776930

[zoaf052-B31] Klingenberg CP , 2011. Morphoj: an integrated software package for geometric morphometrics. Mol Ecol Resour 11(2):353–357. 21429143 10.1111/j.1755-0998.2010.02924.x

[zoaf052-B32] Klingenberg CP, Leamy LJ, 2001. Quantitative genetics of geometric shape in the mouse mandible. Evolution 55(11):2342–2352. 11794792 10.1111/j.0014-3820.2001.tb00747.x

[zoaf052-B33] Klingenberg CP, Marugán-Lobón J, 2013. Evolutionary covariation in geometric morphometric data: analyzing integration, modularity, and allometry in a phylogenetic context. Syst Biol 62(4):591–610. 23589497 10.1093/sysbio/syt025

[zoaf052-B34] Kovaleva VY, Pozdnyakov AA, Litvinov YN, Efimov VM, 2019. Estimation of the congruence between morphogenetic and molecular-genetic modules of gray voles *Microtus* s.l. Variability along a climatic gradient. Ecol Genet 17(2):21–34.

[zoaf052-B35] Kovaleva VY, Pozdnyakov AA, Litvinov YN, Efimov VM, 2021. Fluctuating asymmetry and morphogenetic correlations of the masticatory surface patterns of m1 in gray voles (Rodentia, Arvicolinae). Biol Bull 48(9):1609–1622.

[zoaf052-B36] Lieberman DE, Krovitz GE, Yatesn FW, Devlin M, Claire MS, 2004. Effects of food processing on masticatory strain and craniofacial growth in a retrognathic face. J Human Evol 46(6):655–677. 15183669 10.1016/j.jhevol.2004.03.005

[zoaf052-B37] Liu F, Dangaria S, Andl T, Zhang Y, Wright AC et al, 2010. β-Catenin initiates tooth neogenesis in adult rodent incisors. J Dent Res 89(9):909–914. 20530729 10.1177/0022034510370090 PMC3148824

[zoaf052-B38] Ma D, Wang X, Guo J, Zhang J, Cai T, 2018. Identification of a novel mutation of RUNX2 in a family with supernumerary teeth and craniofacial dysplasia by whole-exome sequencing: a case report and literature review. Medicine (Baltimore) 97(32):11328. 10.1097/MD.0000000000011328 PMC6133463 30095610

[zoaf052-B39] Mancinelli E, Capello V, 2016. Anatomy and disorders of the oral cavity of rat-like and squirrel-like rodents. Vet Clin North Am Exot Anim Pract 19(3):871–900. 27497210 10.1016/j.cvex.2016.04.008 PMC7110795

[zoaf052-B40] Martinez-Maza C, Montes L, Lamrous H, Ventura J, Cubo J, 2012. Postnatal histomorphogenesis of the mandible in the house mouse. J Anat 220(5):472–483. 22372819 10.1111/j.1469-7580.2012.01488.x PMC3403277

[zoaf052-B41] Maser C, Hooven EF, 1970. Dental abnormalities in *Microtus longicaudus*. The Murrelet 11:11.

[zoaf052-B42] Mavropoulos A, Ammann P, Bresin A, Kiliaridis S, 2005. Masticatory demands induce region-specific changes in mandibular bone density in growing rats. Angle Orthod 75(4):625–630. 16097232 10.1043/0003-3219(2005)75[625:MDIRCI]2.0.CO;2

[zoaf052-B43] Mitteroecker P, Bookstein F, 2011. Linear discrimination, ordination, and the visualization of selection gradients in modern morphometrics. Evol Biol 38(1):100–114.

[zoaf052-B44] Müller J, Clauss M, Codron D, Schulz E, Hummel J et al, 2014a. Growth and wear of incisor and cheek teeth in domestic rabbits (*Oryctolagus cuniculus*) fed diets of different abrasiveness. J Exp Zool A Ecol Genet Physiol 321(5):283–298. 24700486 10.1002/jez.1864

[zoaf052-B45] Müller J, Clauss M, Codron D, Schulz E, Hummel J et al, 2014b. The eternal tooth germ is formed at the apical end of continuously growing teeth. Arch Oral Biol 50(2):153–157. 10.1016/j.archoralbio.2004.09.008 15721143

[zoaf052-B46] Nikol'sky VS , 1997. General principles of biomechanics in jaw apparatus of mammals. Zool Zhurnal 76(1):94–103.

[zoaf052-B47] Ohshima H, Nakasone N, Hashimoto E, Sakai H, Nakakura-Ohshima K et al, 2005. The eternal tooth germ is formed at the apical end of continuously growing teeth. Arch Oral Biol 50(2):153–157. 15721143 10.1016/j.archoralbio.2004.09.008

[zoaf052-B48] Parés-Casanova PM , 2019. Skull asymmetry in sheep is dominated by right side. J Morphol Anat 3(2):122.

[zoaf052-B49] Parés-Casanova PM, Bravi R, 2014. Directional and fluctuating asymmetries in domestic sheep skulls. J Zool Biosci Res 1(2):11–17.

[zoaf052-B50] Parsons PA , 1992. Fluctuating asymmetry: a biological monitor of environmental and genomic stress. Heredity (Edinb) 68(4):361–364. 1563968 10.1038/hdy.1992.51

[zoaf052-B51] Potapova EG , 2020. Morphofunctional transformations of the jaw muscles in rodent evolution. Biol Bull Rev 10(5):394–406.

[zoaf052-B52] R Core Team , 2024. R: A language and environment for statistical computing Vienna, Austria: R Foundation for Statistical Computing. [cited 2024 January 17]. Available from: https://www.R-project.org/.

[zoaf052-B53] Renaud S, Alibert P, Auffray JC, 2012. Modularity as a source of new morphological variation in the mandible of hybrid mice. BMC Evol Biol 12(1):1–16. 22873779 10.1186/1471-2148-12-141 PMC3506452

[zoaf052-B54] Renvoisé E, Evans AR, Jebrane A, Labruère C, Laffont R et al, 2009. Evolution of mammal tooth patterns: new insights from a developmental prediction model. Evol 63(5):1327–1340. 10.1111/j.1558-5646.2009.00639.x 19187252

[zoaf052-B55] Rohlf FJ . 2016. Morphometrics at suny stony brook. [cited 2024 February 12], Available from: http://sbmorphometrics.org/.

[zoaf052-B56] Söderman F, Van Dongen S, Pakkasmaa S, Merilä J, 2007. Environmental stress increases skeletal fluctuating asymmetry in the moor frog Rana arvalis. Oecologia 151(4):593–604. 17136394 10.1007/s00442-006-0611-0

[zoaf052-B57] Song L , 2017. Calcium and bone metabolism indices. Adv Clin Chem 82:1–46. 28939209 10.1016/bs.acc.2017.06.005

[zoaf052-B58] Štebra O , 1976. Prenatal development of microtine rodents. Acta sci Natur Brno 10:1–41.

[zoaf052-B59] Sugita S, Uchiumi O, Fujiwara K, Niida S, Fukuta K, 1995. Brain deformation caused by hyperplasia molar teeth (macrodonts) in the Japanese field vole (*Microtus montebelli*). Exp Anim 43(5):769–772. 7498346 10.1538/expanim1978.43.5_769

[zoaf052-B60] Vogel P, Liu J, Platt KA, Read R, Vance RV et al, 2015. Malformation of incisor teeth in Grem2−/− mice. Vet Pathol 52(1):224–229. 24686385 10.1177/0300985814528218

[zoaf052-B61] Wang XP, Suomalainen M, Felszeghy SZ, Zelarayan LC, Alonso MT et al, 2007. An integrated gene regulatory network controls stem cell proliferation in teeth. PLoS Biol 5(6):1324–1333. 10.1371/journal.pbio.0050159 PMC1885832 17564495

[zoaf052-B62] Weijs WA , 1975. Mandibular movements of the albino rat during feeding. J Morphol 145(1):107–124. 1111422 10.1002/jmor.1051450107

[zoaf052-B63] Whitlock MC , 2003. Fixation probability and time in subdivided populations. Genetics 164(2):767–779. 12807795 10.1093/genetics/164.2.767 PMC1462574

[zoaf052-B64] Yamada K, Kimmel DB, 1991. The effect of dietary consistency on bone mass and turnover in the growing rat mandible. Arch Oral Biol 36(2):129–138. 1711839 10.1016/0003-9969(91)90075-6

[zoaf052-B65] Yiğit N, Çetintürk D, Çolak E, 2017. Phylogenetic assessment of voles of the Guentheri group (Mammalia: Microtus) in Turkish Thrace and western Anatolia. Eur Zool J 84(1):252–260.

[zoaf052-B66] Zakharov VM, Chubinishvili AT, Dmitriev SG, Baranov AS, Borisov V et al, 2000. [Health of the Environment: Practical Evaluation] Moscow: Tsentr Ekolog Politiki Rossii (In Russian).

[zoaf052-B67] Zakharov VM, Yablokov AV, 1997. Developmental homeostasis in natural populations of mammals: phenetic approach-introduction. Acta Theriol 5:8.

[zoaf052-B68] Zamanlu M, Khamnei S, SalariLak S, Oskoee SS, Shakouri SK et al, 2012. Chewing side preference in first and all mastication cycles for hard and soft morsels. Int J Clin Med 5(4):326. PMC3443888 22993653

[zoaf052-B69] Zhou T, Pan J, Wu P, Huang R, Du W et al, 2019. Dental follicle cells: roles in development and beyond. Int J Stem Cells 1:9159605. 10.1155/2019/9159605 PMC6766151 31636679

[zoaf052-B70] Zorenko T . 2021. Effect of social stress on abnormal growth of the incisors of Harting’s vole Microtus hartingi under experimental conditions. In: Abstract book of the 79th International Scientific Conference of the University of Latvia, Riga, Latvia, University of Latvia, 28.

[zoaf052-B71] Zorenko TA , 2022. Communal reproduction of females of two subspecies of the Harting’ vole *Microtus* (Sumeriomys) *hartingi* (Rodentia, Arvicolinae) under experimental conditions. J Zool 101(9):1048–1060.

[zoaf052-B72] Zorenko T, Basov A, Kaija LP, Mitkovska V, 2025. Incisor and mandible anomalies in the Harting's vole Microtus hartingi (Mammalia: Arvicolinae) in Europe. Russian J Theriol 24(1):32–36.

[zoaf052-B73] Zorenko T, Kagainis U, Golenishchev F, Barashkova L, 2023. Geometric morphometrics of the cranium and mandible in social voles of the “Guentheri” group (Arvicolinae: Sumeriomys). Diversity 15(1):83.

[zoaf052-B74] Zorenko T, Kaija LP, 2024. Inbreeding tolerance in two isolated populations of Harting's vole *Microtus hartingi* (Rodentia, Arvicolinae). Turk J Zool 48(2):140–153.

