# Supplementary material for: Morphological diversity of dental and mandibular deformities in Microtus hartingi (Rodentia, Arvicolinae)
Source: Curr Zool. 2025 Aug 13;72(3):307–20. doi: 10.1093/cz/zoaf052 (PMC13290401; doi:10.1093/cz/zoaf052)
Supplement: zoaf052_Supplementary_Data [file zoaf052_supplementary_data.docx]

**Supplementary Figure 1**: Anatomical landmarks and semilandmarks used to assess GM of the labial and lingual side of the hemimandible in Harting’s voles (semilandmarks are indicated with an asterisk).

Description of landmark locations on the labial hemimandible side: 1) the point at the anterior-dorsal border of the incisive alveolus; 2) the point at the most concave point of the diastema; 3) the basal point of the anterior alveolus of the lower premolar; 4) the point at the intersection of the anterior alveolus of the lower premolar and the base of the coronoid process; 5) the point at the tip of the coronoid process; 6) the most concave point of the sigmoid notch; 7) the point at the minimum width on the anterior edge of the articular surface of the condyle; 8) the point of the terminal tip of the mandibular condyle; 9) the point at the posterior edge of the articular surface of the condyle; 10) the most anterior point on the curve of the posterior edge of the mandible; 11) the point at the tip of the mandibular angle; 12) the most dorsal point on the ventral border of the ramus; 13) the most inferior point on the border of ramus inferior to incisor alveolar; 14) the point at the antero-ventral border of the incisive alveolus; 15) the point at half of the distance between landmarks 4 and 5*; 16) the point at half of the distance between landmarks 5 and 6*; 17) the point at half of the distance between landmarks 6 and 7*; 18) the point at half of the distance between landmarks 9 and 10*; 19) the point at half of the distance between landmarks 10 and 11*; 20) the point at half of the distance between landmarks 11 and 12*.

Description of landmark locations on the lingual hemimandible side: 1) the point at the anterior-dorsal border of the incisive alveolus; 2) the point at the most concave point of the diastema; 3) the basal point of the anterior alveolus of the lower premolar; 4) the point at the posterior end of the anterior alveolus; 5) the point at the tip of the coronoid process; 6) the most concave point of the sigmoid notch; 7) the point at the minimum width on the anterior edge of the articular surface of the condyle; 8)the point of the terminal tip of the mandibular condyle; 9) the point at the posterior edge of the articular surface of the condyle; 10) the most anterior point on the curve of the posterior edge of the mandible; 11) the point at the tip of the mandibular process; 12) the point at the intersection of the third molar alveolus and the ramus; 13) the most inferior point on the border of ramus inferior to incisor alveolar; 14) the point at the anteroventral edge of the incisive alveolus; 15) the point at the tip of the third molar alveolus; 16) the point at half of the distance between landmarks 5 and 6*; 17) the point at half of the distance between landmarks 6 and 7*; 18) the point at half of the distance between landmarks 9 and 10*; 19) the point at half of the distance between landmarks 3 and 4*; 20) the point at half of the distance between landmarks 4 and 15; 21) the point at half of the distance between landmarks 12 and 15*.

**Supplementary Figure 2:** CVA shape deformation results for mandible datasets of *Microtus hartingi* from Rhodopes. Blue dots indicate landmarks after shape deformation; numbers represent landmark numbers; the scale factor differs and is displayed for each graph. A, C: light-blue outline – normal (N) and dark blue – deformed. B, D: light-blue outline – deformed and dark blue – normal. A: labial left; B: labial right; C: lingual left; D: lingual right hemimandibles


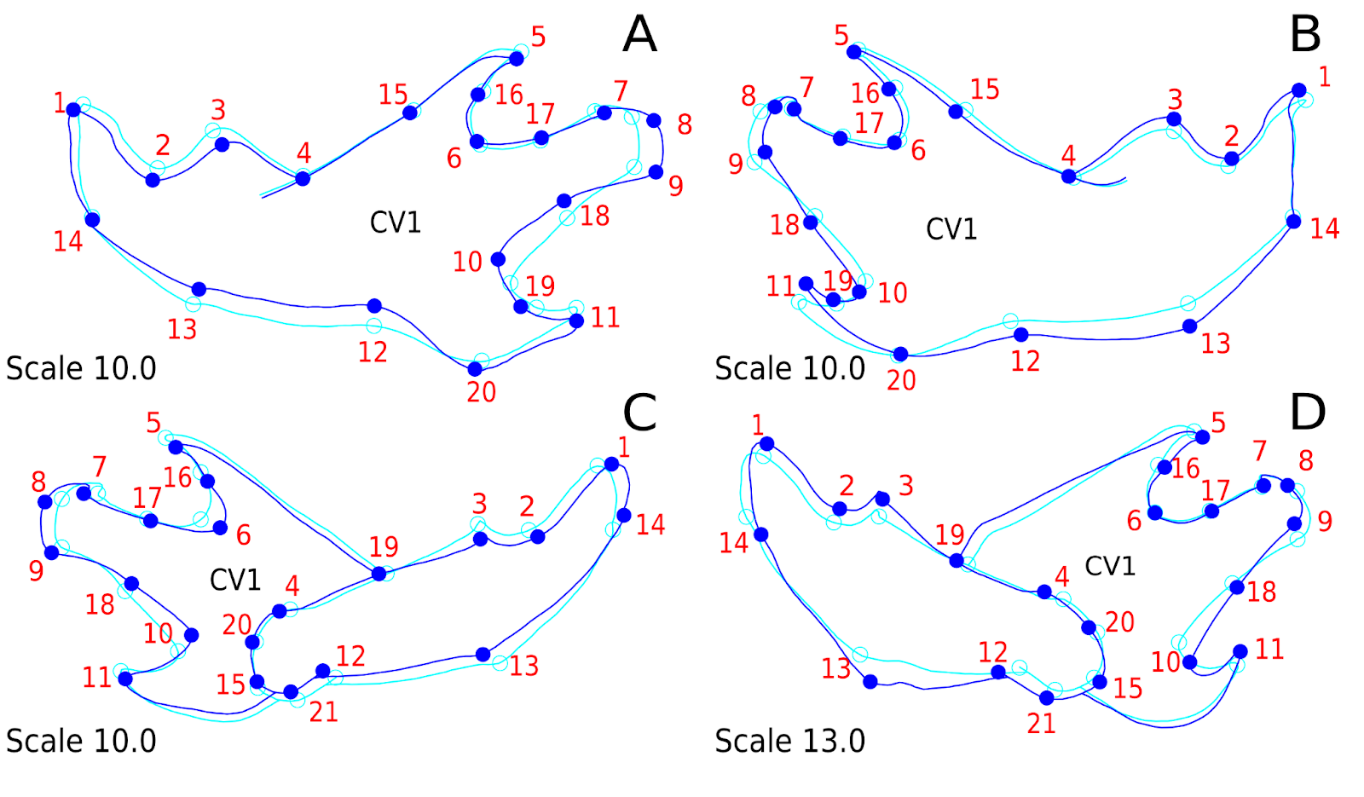


**Supplementary Figure 3**. The CVA shape deformation results for mandible datasets of *Microtus hartingi ankaraensis*. Blue dots indicate landmarks after shape deformation; numbers represent landmark numbers; the scale factor differs and is displayed for each graph. C, D – light-blue outline – deformed and dark blue – normal; A(1), A(2), B – light-blue outline – normal and dark blue – deformed hemimandibles.

Alt 6. Canonical Variate Analysis outline plot of *Microtus hatingi* hemimandibles showing deformation pattern between normal and deformed groups


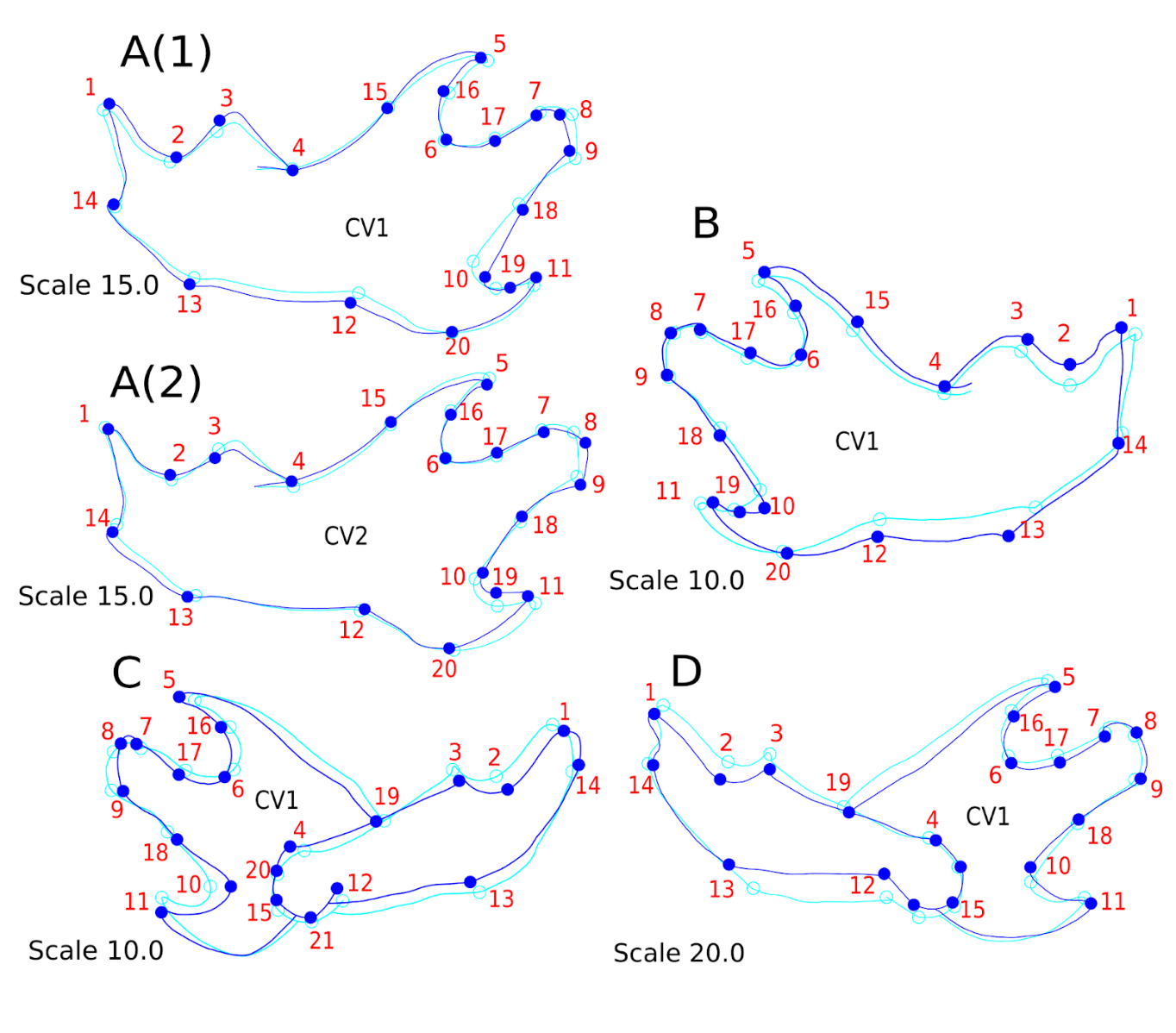


Alt 7. Canonical Variate Analysis outline plot of *Microtus hatingi ankaraensis* hemimandibles showing deformation pattern between initiation of deformation and deformed groups or among normal, initiation of deformation and deformed groups

**Supplementary Table 1.** CVA results for left and right hemimandibles of *Microtus hartingi* comparing normal and deformed hemimandibles (*P* value: permutation *P* value; boldfaced: a statistically significant difference). Permutation test with 9999 permutations.

| Projection | Side |  |  | Distance | *P* value |  | Distance | *P* value |
| --- | --- | --- | --- | --- | --- | --- | --- | --- |
| Labial | Left |  | Mahalanobis distance | 4.176 | 0.085 | Goodall's F | 3.792 | **<0.001** |
|  |  |  | Procrustes distance | 0.030 | **<0.001** | Pillai's trace | 0.819 | 0.085 |
|  | Right |  | Mahalanobis distance | 4.656 | **0.010** | Goodall's F | 3.070 | **<0.001** |
|  |  |  | Procrustes distance. | 0.026 | **<0.001** | Pillai's trace | 0.849 | **0.009** |
| Lingual | Left |  | Mahalanobis distance | 5.010 | **0.013** | Goodall's F | 3.428 | **<0.001** |
|  |  |  | Procrustes distance | 0.036 | **<0.001** | Pillai's trace | 0.867 | **0.017** |
|  | Right |  | Mahalanobis distance | 4.864 | **0.029** | Goodall's F | 2.824 | **0.003** |
|  |  |  | Procrustes distance | 0.030 | **0.004** | Pillai's trace | 0.859 | **0.030** |

**Supplementary Table 2.** CVA results for left and right hemimandibles of *Microtus hartingi ankaraensis* (N: normal, ID: initiation of deformation, D: deformation). (*P* value: permutation *P* value; boldfaced: a statistically significant difference).  Permutation test with 9999 permutations.

| Projection | Side | Group |  | Distance | *P* value |  | Distance | *P* value |
| --- | --- | --- | --- | --- | --- | --- | --- | --- |
| Labial | Left | N vs ID | Mahalanobis distance | 9.047 | 0.964 | Goodall's F | 4.631 | **<0.001** |
|  |  |  | Procrustes distance | 0.033 | **< 0.001** |  |  |  |
|  |  | ID vs D | Mahalanobis distance | 8.896 | 0.429 |  |  |  |
|  |  |  | Procrustes distance | 0.024 | **0.044** | Pillai's trace | 1.879 | **<0.001** |
|  |  | N vs D | Mahalanobis distance | 11.702 | 0.428 |  |  |  |
|  |  |  | Procrustes distance | 0.039 | **< 0.001** |  |  |  |
|  | Right | ID vs D | Mahalanobis distance | 5.100 | 0.301 | Goodall's F | 2.169 | **0.028** |
|  |  |  | Procrustes distance | 0.042 | **0.032** | Pillai's trace | 1.000 | 0.420 |
| Lingual | Left | ID vs D | Mahalanobis distance | 4.956 | 0.611 | Goodall's F | 0.982 | 0.412 |
|  |  |  | Procrustes distance | 0.019 | 0.410 | Pillai's trace | 1.000 | 0.585 |
|  | Right |  | Mahalanobis distance | 4.946 | 0.821 | Goodall's F | **1.964** | **0.041** |
|  |  |  | Procrustes distance | **0.038** | **0.041** | Pillai's trace | 1.000 | 0.525 |
